# Supplementary material for: PIM kinases facilitate lentiviral evasion from SAMHD1 restriction via Vpx phosphorylation
Source: Nat Commun. 2019 Apr 23;10:1844. doi: 10.1038/s41467-019-09867-7 (PMC6479052; doi:10.1038/s41467-019-09867-7)
Supplement: Supplementary file 1 — Supplementary Information [file 41467_2019_9867_MOESM1_ESM.pdf]

Supplementary Information

PIM kinases facilitate lentiviral evasion from SAMHD1 restriction via Vpx phosphorylation

Miyakawa et al.

Supplementary Figure 1

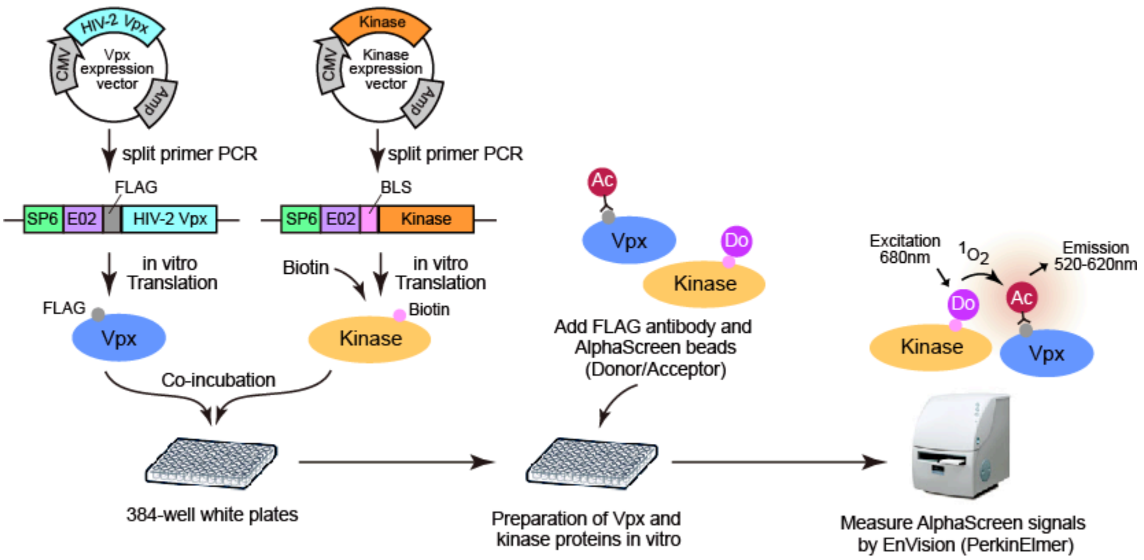

Supplementary Fig. 1. Schematic representation of the AlphaScreen-based screening method.

DNA templates for in vitro transcription were constructed using the host factor (protein kinase) library and split-primer PCR technique, by which the biotin ligation site (BLS) was fused to the N-terminal-coding regions of all genes to allow protein biotinylation. Biotinylated host factors were incubated with FLAG-tagged Vpx protein for 1 hour at 37°C. Subsequently, protein A–conjugated acceptor beads with anti-FLAG antibody and streptavidin-coated donor beads were added and allowed to bind to the tagged proteins. Upon laser excitation, donor beads convert ambient oxygen to singlet oxygen. When both proteins are within 200 nm of each other, singlet oxygen is transferred across the gap to activate acceptor beads and subsequently emit light of 520–620 nm (AlphaScreen signals). If the proteins do not interact (distance >200 nm), no AlphaScreen signal is produced because the singlet oxygen does not transfer from the donor beads to the acceptor beads.

Supplementary Figure 2

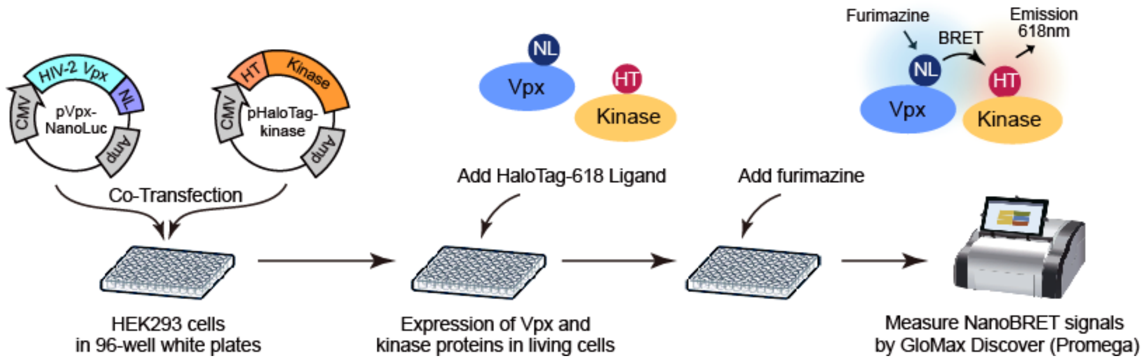

Supplementary Fig. 2. Schematic representation of the NanoBRET-based screening method.

HaloTag-conjugated human kinase expression plasmids were obtained from Kazusa DNA Research Institute. HEK293 cells in 96-well plates were co-transfected with a vector encoding NanoLuc-fused Vpx (1 ng) and each HaloTag-conjugated kinase (100 ng). At 24 hours post-transfection, cells were treated with HaloTag-618 ligand to label the kinase with fluorescein. Twenty-four hours later, the cells were treated with furimazine (substrate of NanoLuc), and the BRET signal was measured on a GloMax Discover instrument.

Supplementary Figure 3

Thr88

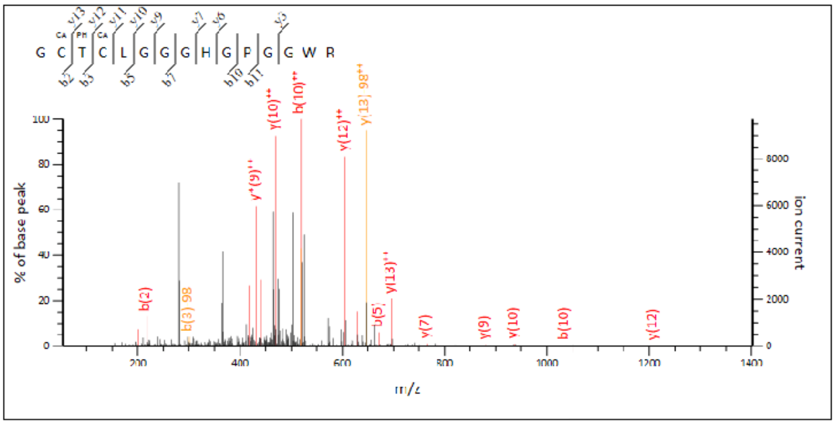

Ser101

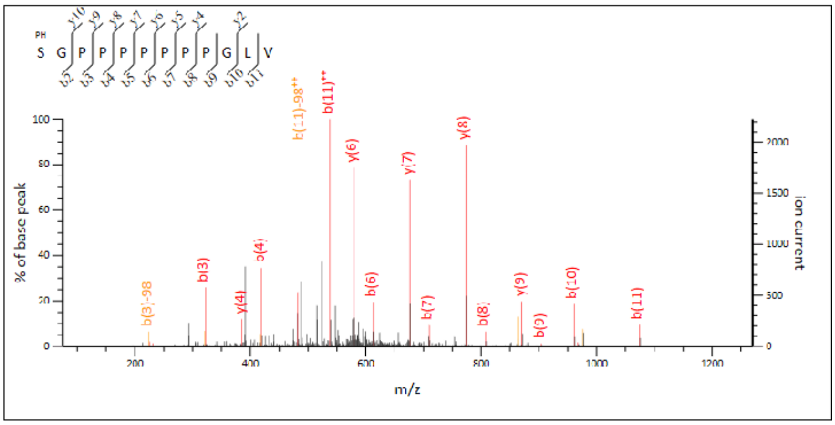

Supplementary Fig. 3. Mass spectra of phosphopeptides, including Thr88 and Ser101.

Y-98 and b-98 designate fragment ions that show neutral loss of phosphoric acid. PH; phosphorylation, CA; carbamidomethylation.

Supplementary Figure 4

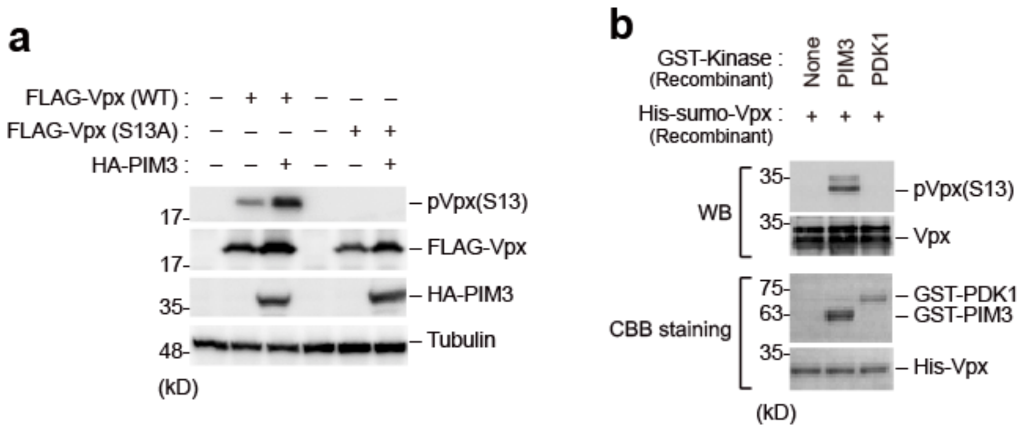

Supplementary Fig. 4. Detection of phosphorylated-Vpx using Phospho-specific antibody.

(**a**) Phospho-specific antibody cannot recognize Vpx-S13A. HEK293 cells (in 12-well plates) were co-transfected with expression vectors encoding FLAG-Vpx and HA-PIM3. Forty-eight hours after transfection, cells were harvested and subjected to immunoblot analysis.

(**b**) PDK1 does not phosphorylate Vpx Ser13. Recombinant Vpx and the indicated kinase proteins (GST-tagged PIM3 or PDK1) were incubated and subjected to immunoblotting with anti-phospho Vpx (Ser13) or anti-Vpx antibodies (top) or CBB staining (bottom). Source data are provided as a Source Data file.

Supplementary Figure 5

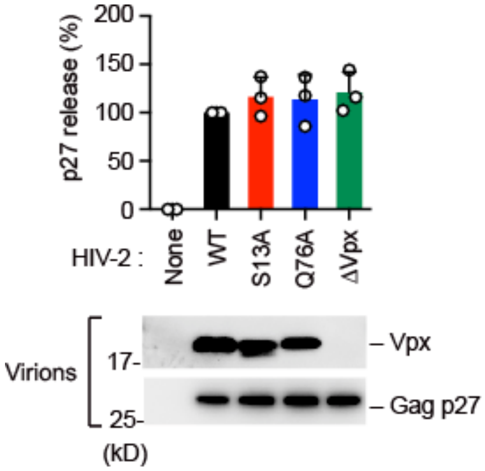

Supplementary Fig. 5. Viral production and Vpx incorporation levels in mutant viruses.

HEK293 cells were transfected with molecular clones carrying the indicated Vpx mutants. After 48 hours, culture supernatants containing virus were collected and subjected to Gag p27 ELISA and immunoblotting. Graph is presented as a mean ± s.d. (n = 3). Source data are provided as a Source Data file.

Supplementary Figure 6

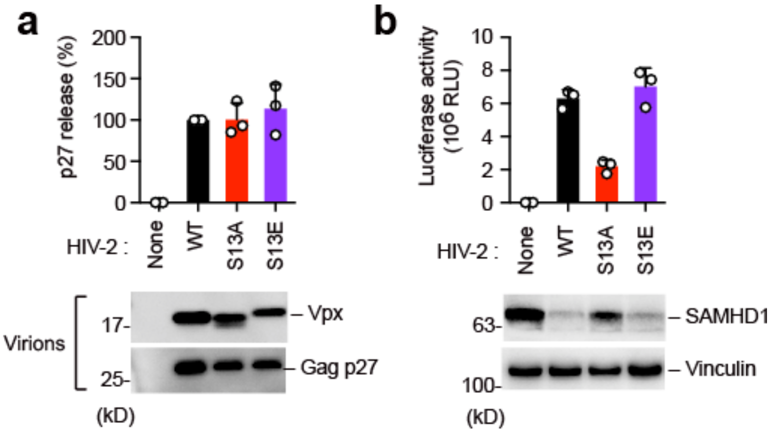

Supplementary Fig. 6. Steady level of viral infectivity of HIV-2 bearing Vpx-S13E.
(**a**) Viral production and Vpx incorporation levels in S13E viruses.
(**b**) Single-cycle HIV-2 infection assays. Monomac6 cells were differentiated into macrophages using PMA, and then infected with the indicated HIV-2 virus harboring a luciferase reporter gene. Forty-eight hours after infection, intracellular luciferase activity was measured. SAMHD1 expression in the indicated cells was analyzed by immunoblotting. All graphs are presented as a mean ± s.d. (n = 3). Source data are provided as a Source Data file.

Supplementary Figure 7

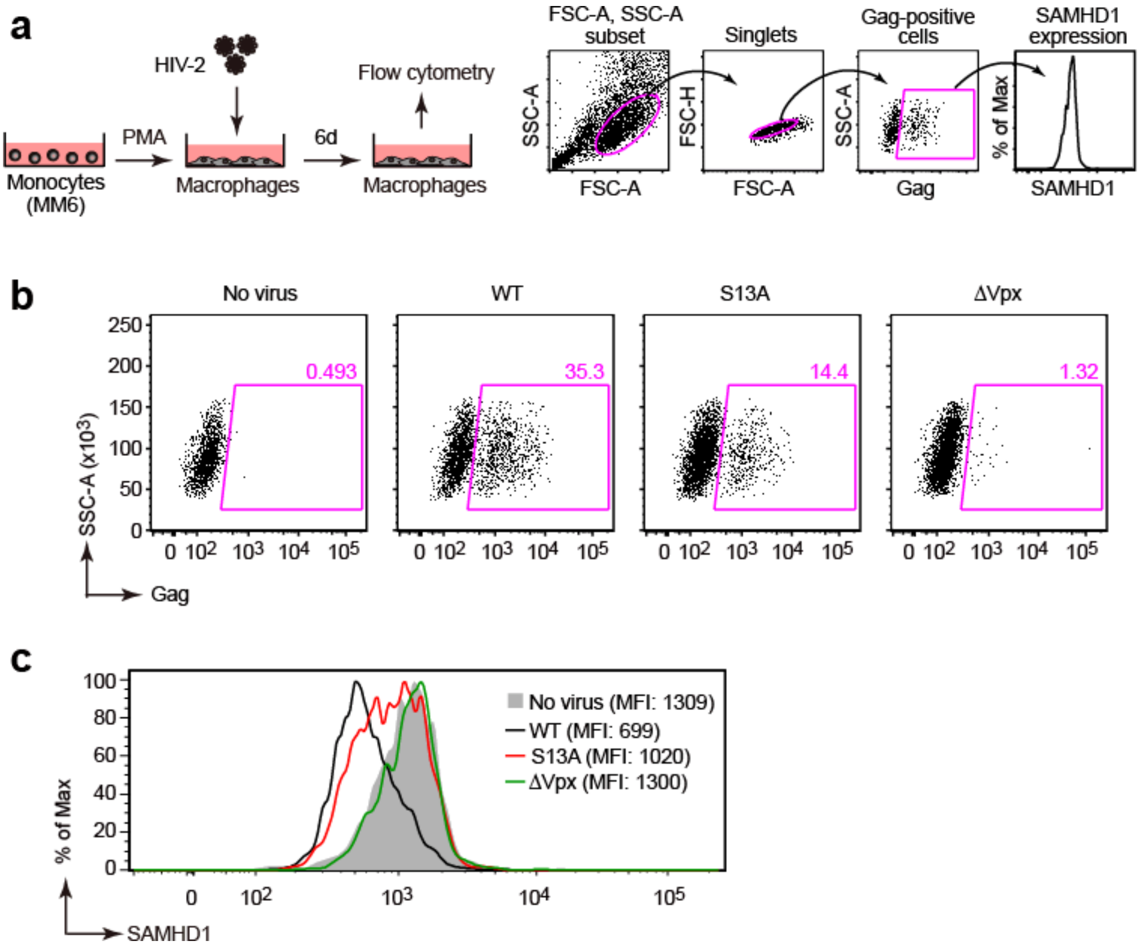

Supplementary Fig. 7. HIV-2 bearing Vpx-S13A exhibits lower viral infectivity with sustained expression of SAMHD1.

(**a**) Schematic representation of flow cytometry-based HIV-2 infection assay used in this study. Briefly, Monomac6 cells were differentiated into macrophages, and then infected with the indicated HIV-2 virus. At 8 days after infection, cells were fixed, permeabilized, and incubated with anti-Gag mouse monoclonal antibody (1:100, NIH AIDS Reagent Program #3537) and anti-SAMHD1 rabbit polyclonal antibody (1:50, Proteintech #12586-1-AP). Cells were then stained with mouse APC- and rabbit FITC-conjugated secondary antibodies (Biolegend) and analyzed using a FACSCanto II instrument (BD Biosciences).

(**b, c**) Gag-positive cells in infected cells (**b**) and SAMHD1 expression in Gag-positive cells (**c**) were calculated by flow cytometry.

Supplementary Figure 8

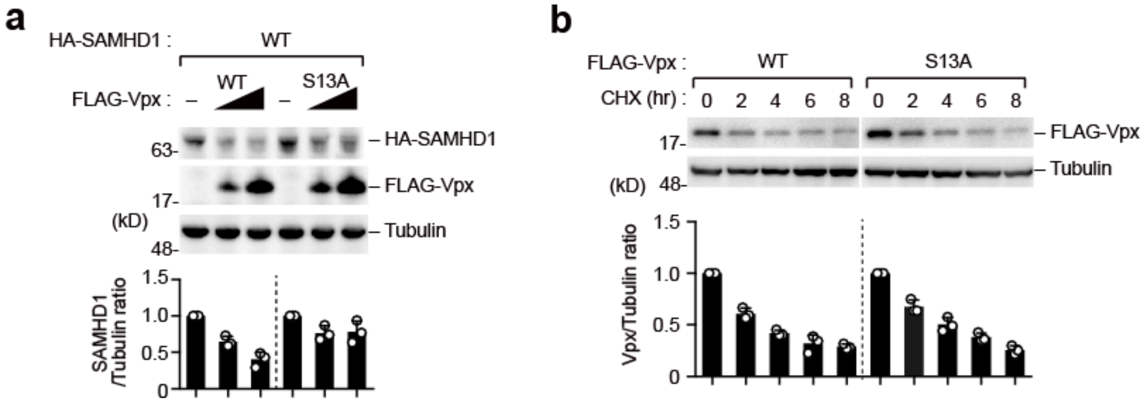

Supplementary Fig. 8. Phosphorylation of Vpx Ser13 does not affect its stability.
(**a**) Transfection-based SAMHD1 degradation assay. HEK293 cells were transfected with plasmids encoding HA-SAMHD1 and the indicated FLAG-Vpx. After 48 hours, cells were harvested and subjected to immunoblot analysis. Bar charts below the blot indicate the levels of SAMHD1, as determined by densitometry.
(**b**) Cycloheximide (CHX) chase assay. HEK293 cells expressing indicated FLAG-Vpx were treated with CHX. Cells were harvested at the indicated time points, and then subjected to immunoblot analysis. Bar charts below the blot indicate the amounts of Vpx, normalized against tubulin, as determined by densitometry. All graphs are presented as a mean ± s.d. (n = 3). Source data are provided as a Source Data file.

Supplementary Figure 9

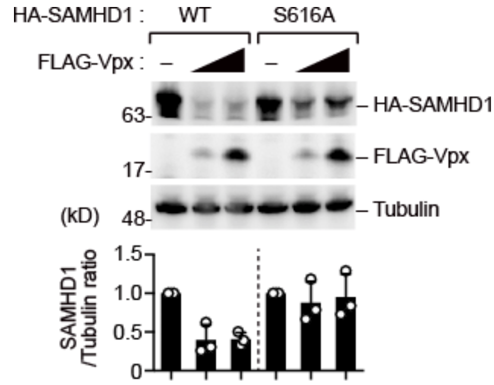

Supplementary Fig. 9. SAMHD1-S616A is resistant to Vpx-induced degradation. HEK293 cells were transfected with plasmids encoding the indicated HA-SAMHD1 and FLAG-Vpx mutants. After 48 hours, cells were harvested and subjected to immunoblot analysis. Bar charts below the blot indicate the levels of SAMHD1, as determined by densitometry. Graph is presented as a mean ± s.d. (n = 3). Source data are provided as a Source Data file.

Supplementary Figure 10

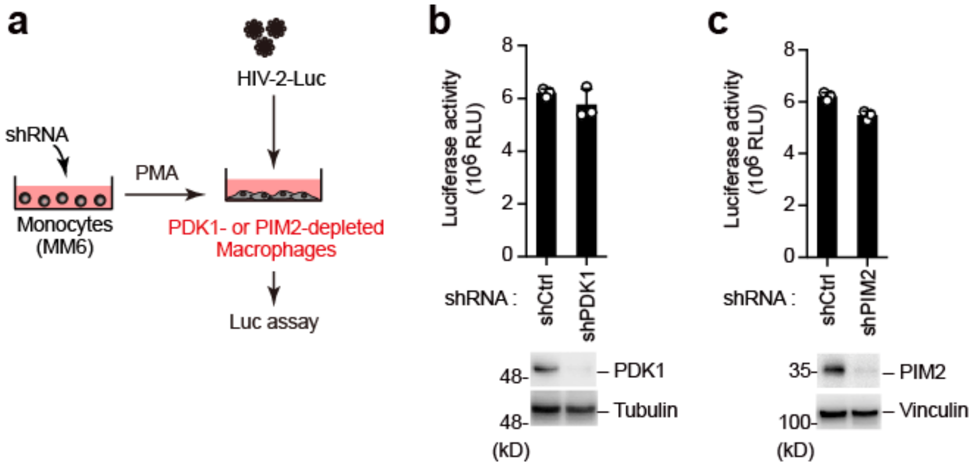

Supplementary Fig. 10. No effects of PDK1 and PIM2 on HIV-2 infection in macrophages.

(**a**–**c**) Schematic representation of the experimental system (**a**). Monomac6 (MM6) cells were stably transduced with shRNAs targeting PDK1 (**b**) or PIM2 (**c**). The cells were then differentiated into macrophages and infected with HIV-2 luciferase (Luc) reporter virus. Forty-eight hours later, the cells were harvested and subjected to luciferase assays to measure HIV-2 infectivity. Expression of PDK1 and PIM2 is also shown. All graphs are presented as a mean ± s.d. (n = 3). Source data are provided as a Source Data file.

Supplementary Figure 11

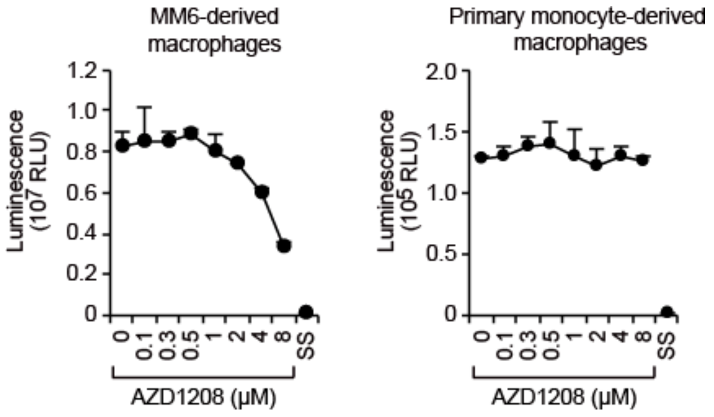

Supplementary Fig. 11. AZD1208 is not cytotoxic at effective concentrations.
Cell viability assay of Monomac6 (MM6) and primary macrophages treated with AZD1208 (0–8 μM) for 48 hours. Staurosporine (SS, 1 μM) was used as a control for induction of cell death. All graphs are presented as a mean ± s.d. (n = 3). Source data are provided as a Source Data file.

Supplementary Table 1

Antibodies	Source (Catalog number)	Dilution
HA	MBL (#M180-3)	1:1000
FLAG	Sigma-Aldrich (#F3165)	1:10000
Myc	Cell Signaling Technology (#2272)	1:1000
GST	Santa Cruz Biotechnology (#sc-138)	1:10000
Vinculin	Sigma-Aldrich (#V9264)	1:1000
α-Tubulin	Sigma-Aldrich (#T6199)	1:10000
SAMHD1	Sigma-Aldrich (#SAB1400478)	1:1000
Vpx	NIH AIDS Reagent Program (#2609)	1:1000
Gag p24	NIH AIDS Reagent Program (#3537)	1:1000
PhosphoVpx(Ser13)	Generated in this study	1:500
PIM1	Cell Signaling Technology (#2272)	1:1000
PIM2	Cell Signaling Technology (#4730)	1:1000
PIM3	Cell Signaling Technology (#4165)	1:1000
PDK1	Proteintech (#10026-1-AP)	1:500
HRP anti-mouse IgG	GE Healthcare (#NA931)	1:10000
HRP anti-rabbit IgG	GE Healthcare (#NA934)	1:10000
HRP anti-mouse IgG (TrueBlot)	Rockland (#18-8817-30)	1:1000

Supplementary Table 1. Antibodies used in this study.
